# Completion norms for 3085 English sentence contexts

**DOI:** 10.3758/s13428-020-01351-1

**Published:** 2020-01-28

**Authors:** Jonathan E. Peelle, Ryland L. Miller, Chad S. Rogers, Brent Spehar, Mitchell S. Sommers, Kristin J. Van Engen

**Affiliations:** 1 Department of Otolaryngology, Washington University in St. Louis, 660 South Euclid, Box 8115, Saint Louis, MO 63110 USA; 2 Department of Neurology, Washington University in St. Louis, St. Louis, MO USA; 3 Department of Psychology, Union College, Schenectady, NY USA; 4 Department of Psychological and Brain Sciences, Washington University in St. Louis, St. Louis, MO USA

**Keywords:** prediction, cloze probability, sentence processing, expectation, response entropy

## Abstract

In everyday language processing, sentence context affects how readers and listeners process upcoming words. In experimental situations, it can be useful to identify words that are predicted to greater or lesser degrees by the preceding context. Here we report completion norms for 3085 English sentences, collected online using a written cloze procedure in which participants were asked to provide their best guess for the word completing a sentence. Sentences varied between eight and ten words in length. At least 100 unique participants contributed to each sentence. All responses were reviewed by human raters to mitigate the influence of mis-spellings and typographical errors. The responses provide a range of predictability values for 13,438 unique target words, 6790 of which appear in more than one sentence context. We also provide entropy values based on the relative predictability of multiple responses. A searchable set of norms is available at http://sentencenorms.net. Finally, we provide the code used to collate and organize the responses to facilitate additional analyses and future research projects.

## Introduction

Language processing exemplifies the interaction between prior knowledge and sensory information, such that an expected stimulus is easier to process than an unexpected stimulus (Howes, 1954; Morton, 1964; Treisman, 1965). In speech perception, varying levels of predictability are associated with different patterns of brain activation in frontal and temporal cortices, reflecting increased input from non-sensory regions in making sense of the auditory stimulus (Blank & Davis, 2016; Obleser, Wise, Dresner, & Scott, 2007; Sohoglu, Peelle, Carlyon, & Davis, 2012). One approach to studying predictability in sentence processing is to compare sentences in which the last word of the sentence is highly predictable (for example, “Art liked milk and sugar in his coffee”) or difficult to predict (for example, “Jamie looked at the bowl”); that is, a dichotomous grouping of high-predictability and low-predictability sentences. Comparing high versus low context sentences has been a productive approach to understanding sentence processing (Bilger, Nuetzel, Rabinowitz, & Rzeczkowski, 1984; Kalikow, Stevens, & Elliott, 1977). However, a potentially more detailed understanding might be obtained by examining predictability in a continuous, rather than categorical, manner.

One way to assess the predictability of a word in a sentence is a cloze procedure in which the sentence missing a target word is presented to a group of participants, and participants are asked to make their best guess as to what the target word was (Taylor, 1953). For instance, using an example sentence from the prior paragraph, “Art liked milk and sugar in his ________”. Although “coffee” would likely be the most frequent response, some participants might guess “tea”. Thus, the relative probabilities of potential answers (across the group of participants) can be used as a measure of how likely a particular word is to complete a sentence.

Well-known norms for sentence-final words have been previously produced, including Bloom and Fischler (1980) (329 sentences, 100 respondents). A subset of 119 sentences were normed on different age groups by Lahar et al. (2004), and Hamberger et al. (1996) provided norms for 198 sentences for 100 younger and 30 older adults. Block and Baldwin (2010) provide data on 498 sentences collected from 337 participants. Our goal here was to produce a larger set of sentences to facilitate greater experimenter flexibility in selection of target words and response probabilities.

In addition to the probability of a given target word, the number and strength of the competitors is also important. One way to parsimoniously quantify the perceptual challenge of a target word based on its context is to consider its entropy (Shannon, Weaver, & Burks, 1951). Entropy is relatively low when one response is more probable than others and increases as multiple responses have similar predictabilities. Entropy provides a measure of response uncertainty that can complement the cloze value of a particular target (Lash, Rogers, Zoller, & Wingfield, 2013). That is, whereas cloze values provide estimates of the most probable response, entropy provides an index of the variability across responses.

It is worth noting a distinction between the constraint of the sentence (which is related to our entropy measure: more constraining sentences are likely to generate fewer possible answers) and the predictability of a *particular* target word, given the preceding context. For example, consider the sentence “At night the woman shut the front window and locked the ________.” In this case “door” would likely have a high probability of being guessed for the last word; a word like “refrigerator” is somewhat plausible but would have a low probability of being guessed. A sentence that provides fewer constraints, such as “The woman enjoyed showing people her newly installed ________”, could also plausibly be completed with “refrigerator”, but in this case the lack of specific sentence constraints changes how listeners process the final word. Thus, in both cases, a word with relatively low levels of predictability may be processed differently depending on overall sentence constraints. The distinction between sentence constraint and word predictability has been appreciated in the EEG/ERP literature for some time (DeLong & Kutas, 2016; Federmeier, Wlotko, De Ochoa-Dewald, & Kutas, 2007; Quante, Bolte, & Zwitserlood, 2018; Wlotko, Federmeier, & Kutas, 2012).

By collecting sentence completion norms online, we were able to collect data on a large number of sentences in a relatively short period of time. Our goal is to provide researchers with a large set of sentences and targets that enables them to select subsets that are appropriate for a given research question. We also hope to provide a starting point for other researchers interested in collecting online sentence norms.

## Method

### Materials

Our motivation for these sentence contexts was to experimentally test the effects of varying the predictability of a sentence-final target word. For 615 target words, we attempted to create at least two “low-predictability” and at least two “high-predictability” sentences. Sentences ranged from 8 to 10 words (11–15 syllables) in length and contained 5–6 content words. The predictability was judged subjectively by the researcher constructing the sentence. All of these sentences were reviewed by at least two people and edited if needed (for example, if a grammatical error was identified). Having created sentences that subjectively varied in predictability, we then completed a cloze procedure in which we asked participants to fill in the last word of the sentence. This procedure allowed us to quantify the predictability of sentence-final words. Note that although the original sentences were constructed around a set of putative target words, because these were deleted prior to the cloze procedure we focus on the responses provided by participants.

### Participants

Participants were recruited on Amazon Mechanical Turk. We tested the 3085 sentences in 61 lists of 50 sentences each, and one list of 35 sentences. Participants could complete as many of these lists as they wished. There were 309 unique participants. Participants were paid for their time ($0.75 for each list of 50 sentences, aimed to be competitive with tasks of similar duration at the time the job was posted) and underwent an informed consent procedure approved by the Washington University Institutional Review Board.

### Procedure

Sentences were presented visually with the last word replaced by a blank. Participants were given the following written instructions:

Please do your best to complete the sentences by typing in the first word that enters your mind. We are looking for the first word that comes to mind, not the most interesting response.

For each sentence, we requested sentence completion from 105 participants, as our aim was at least 100 useable responses for each sentence. After exclusions (see below) we collected 326,673 responses.

### Analysis

Code for analyses is available from https://github.com/jpeelle/sentence-prediction. The deidentified raw data, norms, summary scripts, and full set of results reported here are available from https://osf.io/jnhqb/, and searchable via a web interface at http://sentencenorms.net. Output files contain summarized responses (each unique response to a sentence expressed as a proportion) in both plain text (Markdown; https://daringfireball.net/projects/markdown/) and tab-separated formats, with one sentence per row.

For each sentence, we tallied all of the unique responses provided by participants, and for each response calculated the proportion of participants who provided it. This number is the cloze probability and reflects the likelihood of a particular response being used to complete a sentence given the preceding sentence context.

Mis-spellings and pluralization presented significant challenges. In our initial testing, automated approaches (e.g., using a dictionary) missed a large number of items. Thus, we went through each response by hand and created a file of replacements that were completed prior to response frequencies being calculated. For example, in our analysis “bee hive”, “beehive”, and “behive” were all counted as the same response. Differences in tense or pluralization were combined when appropriate, and responses judged to be typos were corrected. For example, for the sentence “The hunter took the antlers from the dead __________”, the response “deet” was changed to “deer” (a real word that fit the context and matched a common response given by other participants). Because our particular goal involved speech perception, when in doubt we made decisions based on phonological similarity. The list of replacements can be seen in the “replacements.csv” file provided with the code. We made a total of 3334 replacements (approximately 1% of the responses, with at least one replacement in 1691 of the sentences).

In addition to the number of unique responses and their respective probabilities, we calculated entropy (*H*) using the number of different responses given and the probability distribution of the responses:

$$ H=-\sum \limits_{i=1}^np\left({x}_i\right){\log}_bp\left({x}_i\right) $$

where *x* is a response, for which there are *n* possible responses (*x*_1_, *x*_2_,…, *x*_*n*_). For each item (*x*_*i*_*)*, there is a probability (*p)* that *x*_*i*_ will occur. The subscript *b* represents the base of the logarithm used; we use base 2 in keeping with the traditional measurement of statistical information represented in bits.

There were a small number of curse words that we decided to exclude from publishing with the norms, but counted in calculations of response characteristics (listed in “censors.csv” provided with the code).

Several participants completed more than one set of sentences, which involved completing more than one set of demographic information. In a small number of cases, participants provided conflicting responses. We went through all responses by hand, and in cases of disagreement we opted for the response that occurred more often.

## Results

### Participants

Of the 309 unique participants, six reported that their native language was not English, and so were excluded from further analyses. The remaining 303 participants ranged in age from 21 to 72 years (mean = 40.2, SD = 11.7). There were 136 males, 163 females, one other, and three who left the question blank or declined to indicate sex. The range of lists completed by a single person was 1–62 (mean = 21.4, SD = 20.40). All of the included participants reported themselves to be native speakers of English living in the United States.

### Sentence completion norms

Responses for two example sentences are shown in Table 1. These examples demonstrate variability in both the number of responses (11 vs. 6), the likelihood of the most common response (0.43 vs. 0.94), and response entropy (2.66 vs. 0.48).

**Table 1.** Responses for two example sentences

Sentence	Completion	Proportion
He hated bees and feared encountering a	hive	0.43
	swarm	0.19
	bee	0.09
	nest	0.08
	wasp	0.06
	beehive	0.04
	sting	0.04
	stinger	0.03
	hornet	0.02
	disease	0.01
	yellowjacket	0.01
	No response	0.01
The baby’s face puckered when she ate something	sour	0.94
	salty	0.01
	bitter	0.01
	slimy	0.01
	tart	0.01
	sweet	0.01
	No response	0.01

**Fig. 1:**
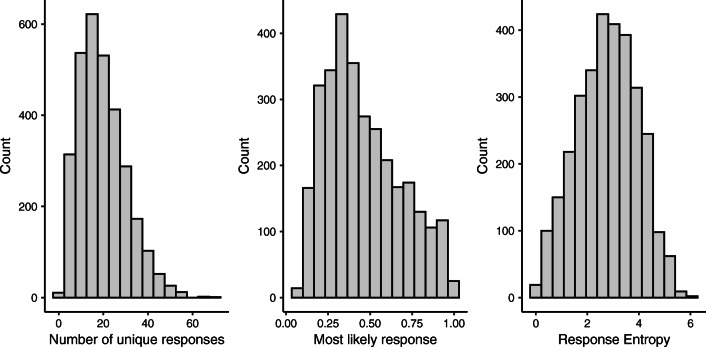
Distribution of response characteristics for each sentence, including the number of unique responses, the most common responses for each sentence, and response entropy

Figure 1 shows the distribution of the number of total responses, probability of the most common response, and response entropies across all 3085 sentences.

**Fig. 2:**
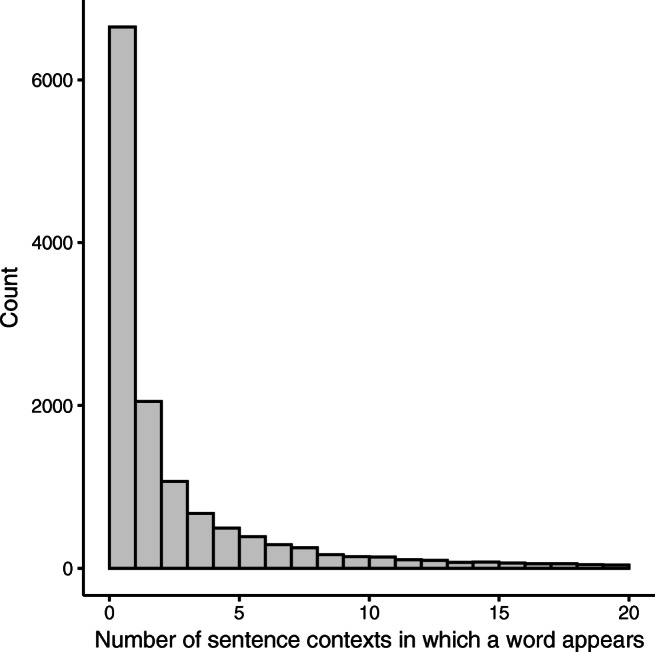
Distribution of the number of sentence contexts in which each target word appeared

Finally, we examined the words provided by participants (which we refer to as target words based on a likely use in an experiment). There were 13,438 unique targets provided. The distribution of how many sentences each word appears in is shown in Fig. 2. Of the response, 6790 target words occurred in more than one sentence context. For example, the word “song” appeared in:

- “To honor her deceased uncle, the niece sang a __________” (cloze probability 0.81)
- “The confident man claimed he could produce a hit __________” (cloze probability 0.50), and
- “The competition started when they heard the __________” (cloze probability 0.03).

## Discussion

The goal of this study was to provide a large set of sentence contexts associated with a range of possible sentence-final words in a format that facilitates selecting subsets for a variety of experimental designs. We calculated sentence completion norms and response entropy calculations for 3085 sentences, each of which was completed by at least 100 participants. These norms allow researchers to select sentences that have words varying in predictability and entropy, or, given a set of target words, to identify sentence contexts for which the word is a plausible ending.

One of our motivations in making the analysis code available is to facilitate analyses by researchers who may prefer alternative analysis strategies. There are several parts of the process requiring subjective decisions (for example, whether to combine “similar” responses); automating several stages of the process makes it more possible for researchers to reproduce the norms using different approaches than we have, or indeed, to perform a similar analysis on a new set of norms.

It is important to note that Lahar et al. (2004) show that the recency of norms are collected may matter, as might the age of the respondents. Fortunately, our participants showed a relatively good range of ages. However, our hope is that by providing a semi-automated process for collating and scoring responses we have facilitated looking at these issues in future samples to control for cohort effects. In addition, we did not include any sentences from prior studies, and so are unable to compare results from different cohorts. Future studies might benefit from including sentences from prior norming studies to allow cross-study comparison in a common set of sentences.

## References

[CR1] Bilger RC, Nuetzel JM, Rabinowitz WM, Rzeczkowski C (1984). Standardization of a test of speech perception in noise. J Speech Hear Res.

[CR2] Blank H, Davis MH (2016). Prediction errors but not sharpened signals simulate multivoxel fMRI patterns during speech perception. PLoS Biology.

[CR3] Block CK, Baldwin CL (2010). Cloze probability and completion norms for 498 sentences: behavioral and neural validation using event-related potentials. Behav Res Methods.

[CR4] Bloom PA, Fischler I (1980). Completion norms for 329 sentence contexts. Memory and Cognition.

[CR5] DeLong KA, Kutas M (2016). Hemispheric differences and similarities in comprehending more and less predictable sentences. Neuropsychologia.

[CR6] Federmeier KD, Wlotko EW, De Ochoa-Dewald E, Kutas M (2007). Multiple effects of sentential constraint on word processing. Brain Res.

[CR7] Hamberger MJ, Friedman D, Rosen J (1996). Completion norms collected from younger and older adults for 198 sentence contexts. Behavior Research Methods Instruments & Computers.

[CR8] Howes D (1954). On the interpretation of word frequency as a variable affecting speed of recognition. Journal of Experimental Psychology.

[CR9] Kalikow DN, Stevens KN, Elliott LL (1977). Development of a test of speech intelligibility in noise using sentence materials with controlled word predictability. Journal of the Acoustical Society of America.

[CR10] Lahar CJ, Tun PA, Wingfield A (2004). Sentence-final word completion norms for young, middle-aged, and older adults. Journal of Gerontology: Psychological Sciences.

[CR11] Lash A, Rogers CS, Zoller A, Wingfield A (2013). Expectation and entropy in spoken word recognition: Effects of age and hearing acuity. Experimental Aging Research.

[CR12] Morton J (1964). The Effects of Context on the Visual Duration Threshold for Words. Br J Psychol.

[CR13] Obleser J, Wise RJS, Dresner MA, Scott SK (2007). Functional integration across brain regions improves speech perception under adverse listening conditions. Journal of Neuroscience.

[CR14] Quante L, Bolte J, Zwitserlood P (2018). Dissociating predictability, plausibility and possibility of sentence continuations in reading: evidence from late-positivity ERPs. PeerJ.

[CR15] Shannon CE, Weaver W, Burks AW (1951). The Mathematical Theory of Communication. Philosophical Review.

[CR16] Sohoglu E, Peelle JE, Carlyon RP, Davis MH (2012). Predictive top-down integration of prior knowledge during speech perception. Journal of Neuroscience.

[CR17] Taylor WL (1953). "Cloze procedure": A new tool for measuring readability. Journalism Quarterly.

[CR18] Treisman AM (1965). Effect of verbal context on latency of word selection. Nature.

[CR19] Wlotko EW, Federmeier KD, Kutas M (2012). To predict or not to predict: Age-related differences in the use of sentential context. Psychology and Aging.

